# “I Go Outdoors for Activities Every Day”: Go-Along With Seniors With Slow Walking Speeds to Explore Environmental Factors Influencing Mobility

**DOI:** 10.3389/ijph.2024.1607033

**Published:** 2024-06-04

**Authors:** Yanchuan Mou, Yu Qin, Shaofei Niu

**Affiliations:** ^1^ School of Architecture and Urban Planning, Chongqing University, Chongqing, China; ^2^ Key Laboratory of Monitoring, Evaluation and Early Warning of Territorial Spatial Planning Implementation, Ministry of Natural Resources, Chongqing, China; ^3^ College of Architecture and Environment, Sichuan University, Chengdu, Sichuan, China

**Keywords:** seniors, slow walking speed, mobility, go-along interview, walking barriers

## Abstract

**Objectives:**

This study aims to: 1) Explore the mobility experiences of seniors with slow walking speeds (SSWS) in urban neighborhoods; and 2) Investigate their environmental barriers and supports.

**Methods:**

Go-along interviews were conducted with 36 SSWS residing in urban neighborhoods of Chongqing City, China. The mobility patterns and built environment factors influencing their mobility were revealed through cartographic analysis and thematic analysis.

**Results:**

SSWS primarily focused their activities within a 400-meter radius of their homes. Built environment themes included topography, neighborhood services, sidewalks, seating, traffic safety, weather, greenery, and lighting. Significant mobility barriers included long stairs, steep slopes, fast-moving objects on sidewalks, road crossings, and fast traffic. Available handrails, nearby food-service places, ample seating, and greenery were identified as supportive factors for their mobility.

**Conclusion:**

This study stands out as the first to specifically examine the mobility of SSWS within the built environment. We suggest that SSWS should be taken into account when establishing a benchmark for general design frameworks. These improvements not only contribute to the mobility of slow walkers but also have positive impacts on the broader population.

## Introduction

As of 2020, the world’s population of people aged 60 and older stands at 1 billion, and this number is projected to double by 2050 [1]. This demographic shift highlights the increased urgency to understand the needs, challenges, and factors contributing to a successful and positive aging experience.

Mobility is defined as the ability to move independently and directly correlates with physical activity, independence, social participation, and overall health [2, 3]. Mobility limitations are increasingly prevalent among older adults, affecting approximately 35% of those aged 70 and the majority of those over 85 years [4]. Walking speed stands out as the most consistently reported mobility measure, particularly in the context of older adults [5, 6]. A large-sample size study has demonstrated that a slower walking speed is associated with lower survival rates in older adults [7]. The average walking speed observed in community-dwelling of active older adults is approximately 1.2 m/s, with a commonly used fixed cut-off of 0.8 m/s to define slow walking speed [8, 9].

Numerous articles have explored how the neighborhood environment supports physical activities like walking and leisure-time activity [10–17]. However, most of these studies have focused on the general older population. It is crucial to acknowledge that individuals with mobility limitations may exhibit different daily activity-travel patterns, given that their mobility constraints significantly influence their choices. A few studies have focused on older adults with mobility disabilities using wheelchairs [18, 19], there is a scarcity of research specifically addressing slow walkers. It could be inferred that the act of walking, whether with or without the assistance of walking aids, represents a distinct mode of mobility compared to using a wheelchair. Consequently, this population has been largely overlooked in research on mobility limitations in older adults. Therefore, it is crucial to investigate the impact of the environment on the mobility of seniors with slow walking speeds (SSWS) and understand their walking barriers and concerns.

This study aims to understand the focal points, barriers, and potential areas for improvement in the built environment by exploring the mobility experiences of SSWS. Urban areas present a complex built environment characterized by diverse physical limitations, including long distances, steep gradients, obstacles on sidewalks, fast-moving cars, and heavy traffic [17, 38]. The go-along interview is an innovative approach for obtaining contextualized perspectives by conducting mobile interviews, wherein fieldworkers accompany individual informants on outings in their everyday environment [20–22]. The study aims to gain a deeper understanding of the built environment’s barriers and supports to mobility for SSWS through the use of go-along interviews. The research questions of this study include: 1) Exploring the mobility experiences of SSWS in urban neighborhoods; and 2) Identifying recurring physical environmental themes and factors potentially influencing their mobility and physical activity. Accordingly, this paper sheds light on environmental features that may enhance mobility, drawing insights from an empirical study of SSWS residing in urban communities in Chongqing, China.

## Methods

### The Go-Along Interview Method

Go-alongs, which combine participant observation with interviewing, provide specific advantages in investigating the importance of place in daily life experiences [22]. The level of researcher involvement during the movement process and the mode of transportation may vary depending on the research objectives [23]. Unlike traditional methods that employ decontextualized visual material or interviews, the go-along approach provides a natural forum for participants to express their thoughts on the community [20]. The go-along interviews provide insights into how participants engage with their neighborhoods in often subtle and non-verbalized ways—socially, physically, and emotionally [24]. Consequently, the go-along interview is gaining attention as an approach particularly useful when understanding relies heavily on peoples’ experiences within their local residential context [20, 21].

### Study Area

Chongqing is one of the most densely populated cities in China, and faced the challenge of a rapidly aging population, with individuals aged 60 and above constituting 21.87% of the population in 2020, making it one of the cities in China with a notably severe aging population. For data collection, the Tuwan neighborhood, a densely populated neighborhood in a typical old urban area, was selected. The residential landscape within the study area is diverse, encompassing old 5–7 story residential buildings (lower housing price) and new high-rise condominiums (higher housing price), representing a mixed-use neighborhood that mirrors the socioeconomic diversity among its residents.

### Recruitment and Participants

**FIGURE 1:**
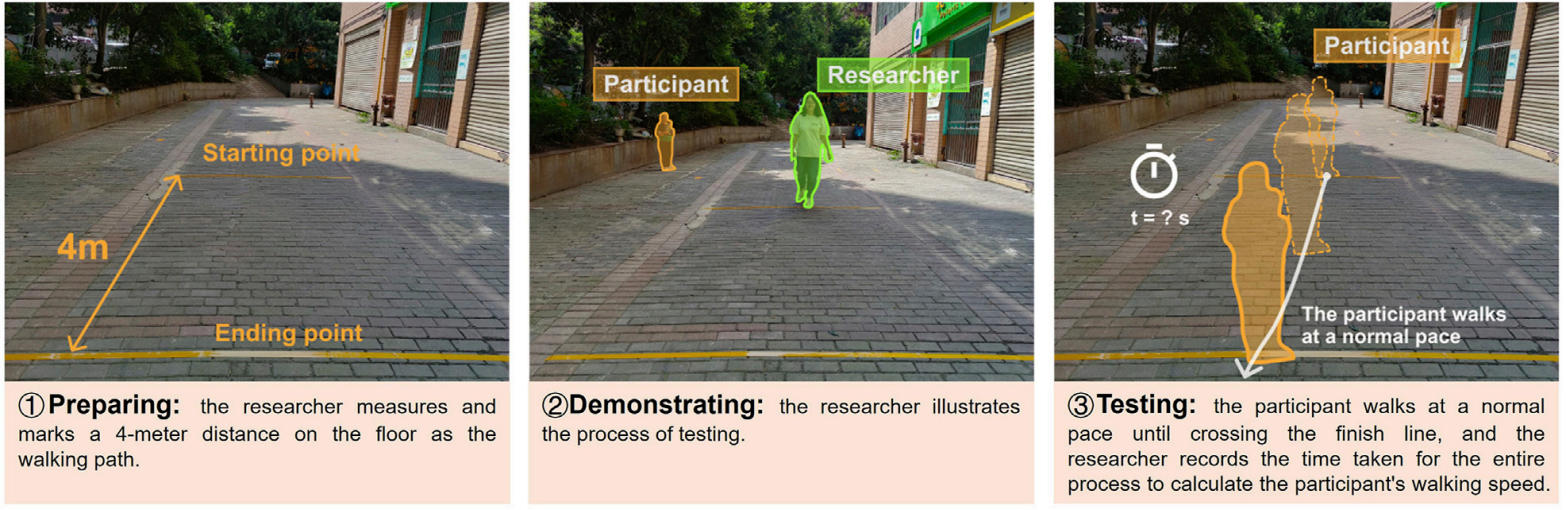
Walking speed test based on the 4-meter walk test (Chongqing, China. 2024).

In general, 4, 6, and 10 m are commonly used for short-distance walk tests for older adults [25]. In this study, the 4-meter walk test (4mWT) was chosen due to its simplicity and feasibility, allowing non-professional personnel to conduct the test [26, 27]. The procedure involves three specific steps, and have been detailed in Figure 1.

A street-intercept method, conducted by two research assistants, was employed to recruit participants. To be included, participants had to be over 60 years old, residing in the community and meeting the criteria for slow walking (<0.8 m/s). Participants were not instructed to follow predetermined routes or confine their movements within the study area. Instead, to gather comprehensive information and empower participants, this study adopted an unstructured approach. During the interviews, participants assumed the role of tour guides, leading the walk and providing insights into their regular routes. The interviews started at the entrance of the participant’s home and concluded at a neighborhood destination, providing insights into participants’ out-of-home behaviors. Each interview lasted a maximum of 60 min, concluding either when the participant remained at the destination for more than 30 min or when the go-along interview reached 60 min.

### Data Collection and Analysis

**FIGURE 2:**
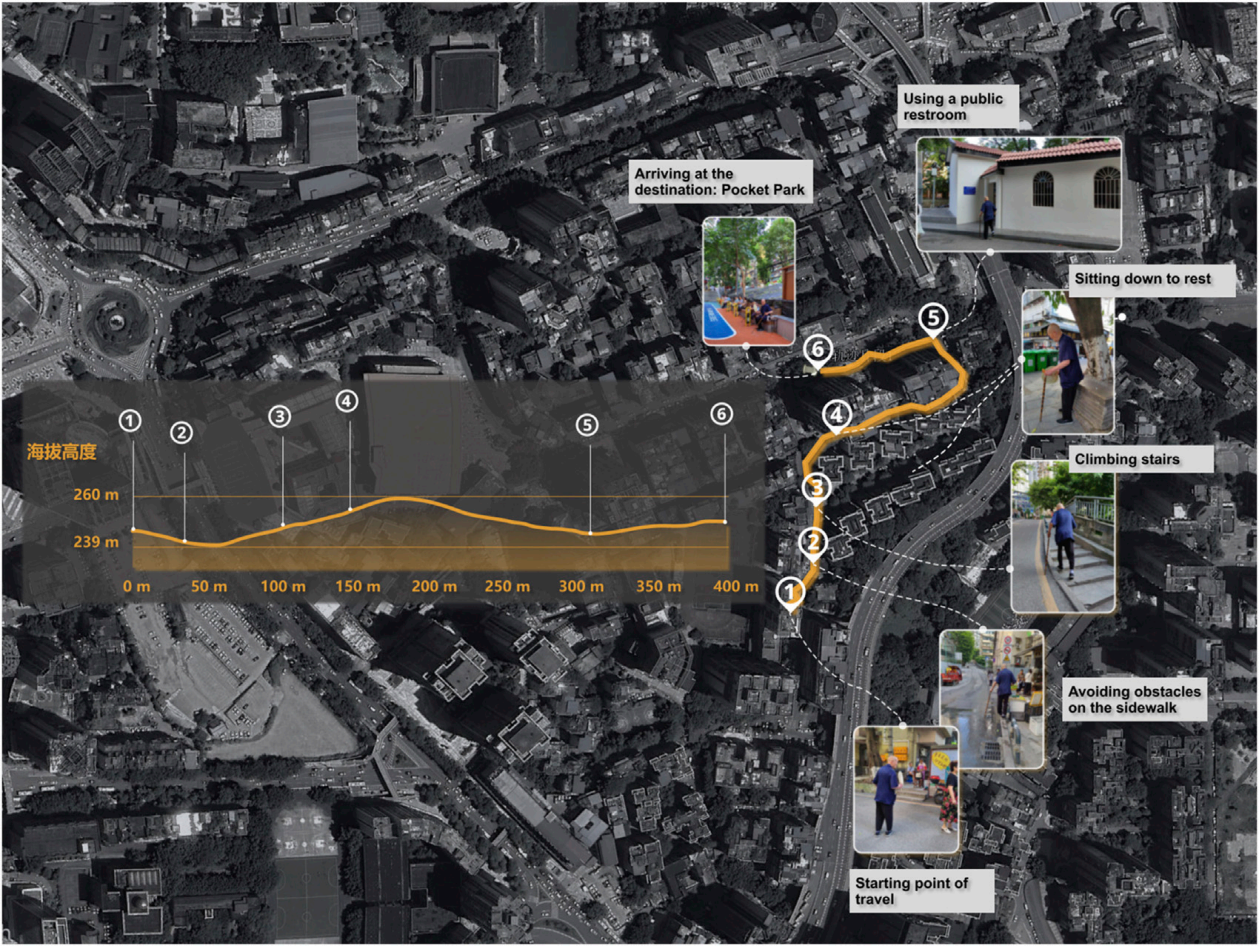
Example of a participant’s go-along interview (Chongqing, China. 2024).

The Geographic Positioning System (GPS) devices were used to capture the routes of the go-along interviews. Throughout the go-alongs, researchers observed participants and documented their physical reactions to the surrounding environment [24]. Participants were asked to share insights into their daily routes and physical activity behavior, including reasons for selecting specific routes. Additionally, they provided comments and recounted their experiences regarding the walking environment and mobility. In instances where participants identified barriers or support during the interview, researchers took photos or videos and had participants narrate the experience. The interviews were recorded, transcribed, and synchronized with spatial and visual data generated by the routes and photos, providing a means to integrate narratives from qualitative interviews alongside quantitative spatial data (Figure 2).

The go-along interviews were conducted between July and October 2023, spanning weekdays and weekends from 8:00 a.m. to 7:00 p.m. A total of 36 individuals participated in the study, contributing 36 spatial routes, 36 interview records, and 258 photos. All participants signed informed consent forms prior to their participation.

The cartographic analysis of spatial data aimed to understand the movement patterns of SSWS within their neighborhoods, including their route preferences and daily activity-travel patterns. Data collected from the GPS devices were exported in spatial forms as KML and processed. To identify the daily activity-travel patterns of SSWS, the Kernel Density Estimation was conducted on the routes and destinations data. Cartographic analysis was performed using ArcGIS 10.2 software.

The thematic analysis of the interview data focused on identifying environmental factors that either facilitate or hinder walking, while also addressing the concerns of SSWS [28]. Micro-environmental factors were especially analyzed due to their significant impact on social life [29]. Two researchers (YM and YQ) coded the transcripts and then proceeded to organize and sort the codes into themes using an inductive approach, which refers to a research methodology involving detailed readings of raw data to derive concepts, themes, or a model through interpretations made by researchers [30]. The NVivo 12 software was employed for the qualitative data analysis.

## Results

### The Participants

Table 1 shows participant characteristics. Thirty-six participants were recruited, with 23 being female. The participants’ ages ranged from 60 to 93 years old, with nearly half of them aged 80 or above. Walking aids were used by 33.3% of the participants. Regarding self-rated health status, 44.4% perceived their health as fair, while 22.2% considered it poor or very poor. Among the participants, 72.2% lived with their spouses or children. All older adults reported traveling for at least 5 days per week, weather permitting. The majority of participants indicated that they had resided in the neighborhood for over 5 years.

**TABLE 1:** Participant characteristics (Chongqing, China. 2024).

Environmental factors	Number (%)
Gender	
Male	13 (36.1%)
Female	23 (63.9%)
Age range	
60∼69	8 (22.2%)
70∼79	13 (36.1%)
80∼89	12 (33.3%)
>90	3 (8.3%)
Use of walking aids when out	
Yes	12 (33.3%)
No	24 (66.7%)
Self-rated health	
Very poor	2 (5.5%)
Poor	6 (16.7%)
Fair	16 (44.4%)
Good	10 (27.8%)
Very good	2 (5.5%)
Living arrangements	
With a partner or children	26 (72.2%)
With others	2 (5.5%)
Alone	8 (22.2%)
The number of days of travel per week	
<2 days	0 (0%)
3∼4 days	0 (0%)
>5 days	36 (100%)
Length of time living in the neighborhood	
<1 year	0 (0%)
1–5 years	4 (11.1%)
>5 years	32 (88.9%)

### Cartographic Analysis

The destinations related to neighborhood service for SSWS were varied and could be divided into three categories: daily facilities (commercial, institutional, welfare, community center, health and age care), public transport (railway station, bus stop), and public spaces (parks, open space, playground). Almost half of the participants visited two or more destination categories during the go-along interview. Out of the 36 routes, six participants completed round trips, while the others made single trips within the itinerary. Only three participants crossed arterial roads, and two of them reported that they had to cross the road to reach the hospital located on the opposite side.

**FIGURE 3:**
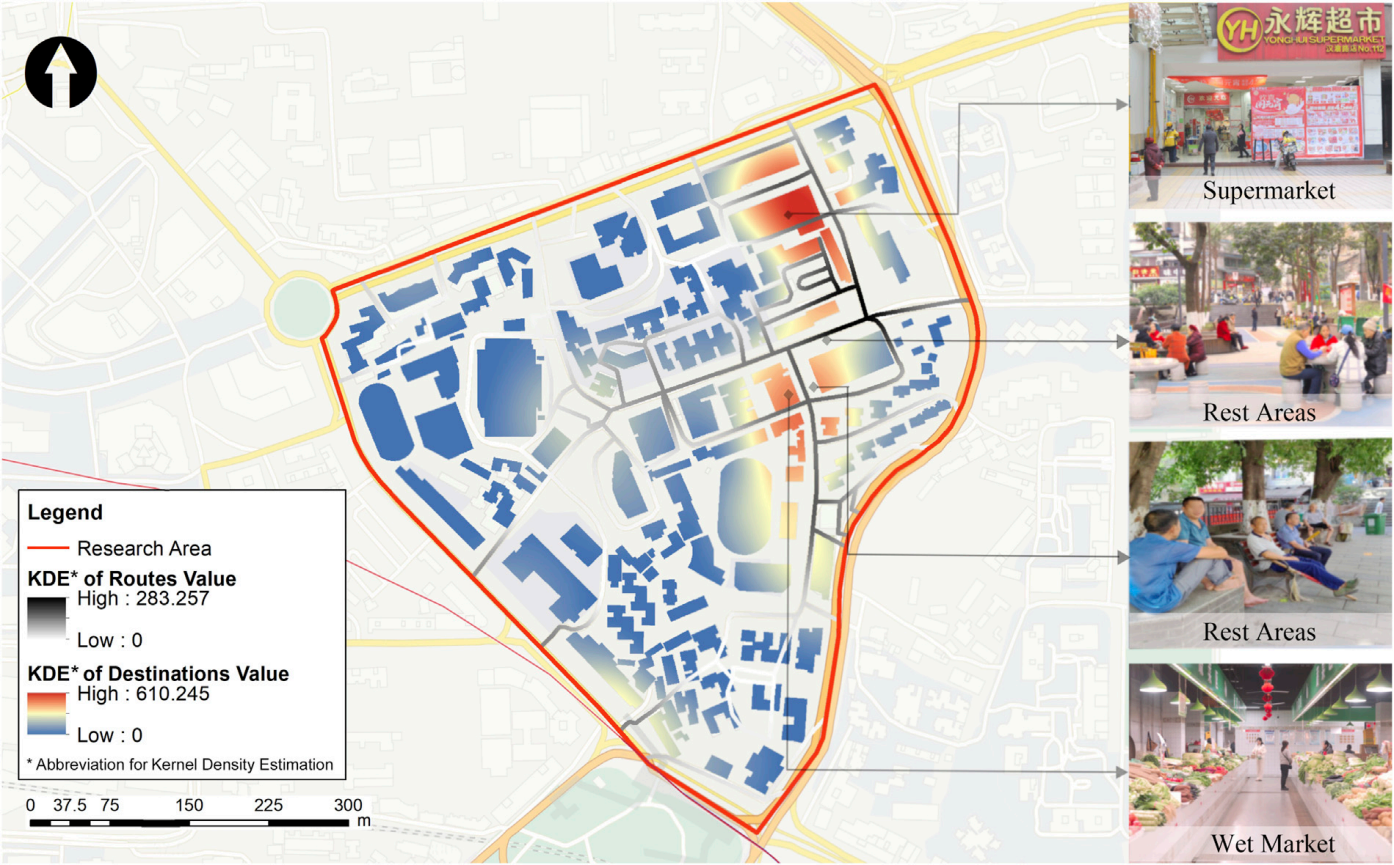
The Kernel density estimation results (Chongqing, China. 2024).

The Kernel Density Estimation of destinations and routes was extracted by masking with road and building maps (Figure 3). The analysis revealed a high-density area on the east side of the study area, indicating consistent travel preferences among SSWS. The paths and destination choices of participants overlapped and intersected at common places.

The walking distance encompasses the distance covered when moving back and forth within a given area. By examining the network distance between the origin and destination of a trajectory, we can determine the extent of life-space mobility for SSWS. For single-destination trips, the range is determined by the network distance between the origin and destination, whereas for multi-destination trips, the range is calculated based on the network distance between the origin and the furthest destination. The results indicate that out of 36 participants, 32 moved within 400 m of their homes (see Supplementary Appendix A). Notably, one participant with the shortest travel distance moved only from their home to the building setback along the street, emphasizing that this setback provided the participant with an opportunity for social interaction.

I got jaundice, and my vision is impaired. I’m afraid to go too far alone because I might bump into someone... I can go down to the square, and I enjoy watching people play cards there. (Participant 16)

### Thematic Analysis

During the go-along interviews, participants discussed environmental factors in their neighborhoods affecting their mobility and walking experiences. Only factors mentioned by two or more participants were included. Eight categories of environmental factors were identified. Table 2 illustrates the percentages of participants who discussed each environmental factor.

**TABLE 2:** Percentages of participants that discussed an environmental category (Chongqing, China. 2024).

Environmental factors	Number (%)
Topographical features	33 (91.7%)
Stair/Step	28 (77.8%)
Ramp	14 (38.9%)
Handrail	14 (38.9%)
Neighborhood services	28 (77.8%)
Daily facilities	18 (50.0%)
Rest areas	16 (44.4%)
Public transport	2 (5.6%)
Overall	10 (27.8%)
Sidewalk environment	18 (50%)
Sidewalk qualities	17 (47.2%)
The behavior of other sidewalk users	2 (5.6%)
Traffic safety	19 (52.8%)
Crossings	14 (38.9%)
Driver’s behavior	5 (13.9%)
Others	2 (5.6%)
Weather	23 (63.9%)
Hot days	16 (44.4%)
Rainy days	9 (25.0%)
Benches and sitting place	19 (52.8%)
The presence of a sitting place	18 (50.0%)
Qualities	3 (8.3%)
Trees and greenery	6 (16.7%)
Lighting	4 (11.1%)

#### Topographical Features

The research area features undulating topography, with height differences primarily connected by stairs ranging from 2 to over 20 steps. While steps do not entirely hinder SSWS from moving around, more than half of the participants (54.5%) who talked about topographical features consider them the primary barrier to their mobility. They expressed feelings of depression and frustration when confronted with lengthy stairs. Few participants (*N* = 3) reported breathlessness when climbing such stairs. Additionally, one participant recounted a personal experience of falling on a long stair, highlighting the challenges they face in navigating such obstacles.

Observations indicated that certain connections have both steps and ramps, with ramps not always chosen over stairs, which is consistent with the findings of Wolfinbarger and Shehab [44]. Two participants expressed that walking on ramps was a more challenging experience than using stairs, especially on steep slopes. Users of assistive devices reported that ramps could pose additional difficulties during descent, particularly in terms of balance and tipping. In addition, one participant mentioned that taking a longer route to avoid stairs could potentially increase the burden of walking.

To go from my home to the wet market, if I take the slope (to avoid stairs), I would have to take a much longer route, which would require more physical effort and more time. I would prefer the stairs. (Participant 28)

Some participants (38.9%) emphasized the importance of feeling safer when using handrails to navigate stairs or walk on ramps. Even if they did not necessarily require handrails, they reported that their presence instilled a sense of security. However, participants also expressed grievances about the frequent unavailability of handrails due to absence, dirtiness, or improper installation.

#### Neighborhood Services

Among the three types of neighborhood services, participants primarily discussed daily facilities and public spaces. Public transportation was rarely mentioned, with only two individuals discussing it, suggesting that those with slow walking speeds utilize public transportation less frequently. The wet market and supermarket were the most commonly mentioned daily facilities. Overall, participants spoke highly of the accessibility of daily facilities, conveniently located within walking distance. However, participants expressed concerns about the environment, citing issues such as slippery floors, sewage overflow, and goods blocking pathways, significantly increasing the risk of falling.

The floor of the market is too slippery, and there is often water on it. I go to the market almost every day, but every time I go, I am nervous and afraid of falling. It would be great if they could replace the floor. (Participant 36)

Some participants (44.4%) reported various types of resting spaces, including pocket parks, squares, building setbacks, and underpasses. Notably, one participant mentioned seeking refuge in the mall to escape the hot weather and pass the time. Additionally, several participants voiced concerns about the community’s lack of resting areas, which not only affects their outdoor activities but also hinders social interactions.

Both nearby pocket parks are filled with people, and there are no empty seats. I go out every day, but there’s nowhere to go. I end up just walking around and coming home. (Participant 11)

#### Sidewalk Environment

The sidewalks in the study area exhibit certain quality issues, including water accumulation, uneven surfaces, and temporary obstructions, which are widely acknowledged as walking barriers. Despite these obstacles, the majority of participants (83.3%) who discussed sidewalk qualities found the sidewalk environment tolerable. Only three participants voiced concerns about slipperiness after rain and maintenance work.

**FIGURE 4:**
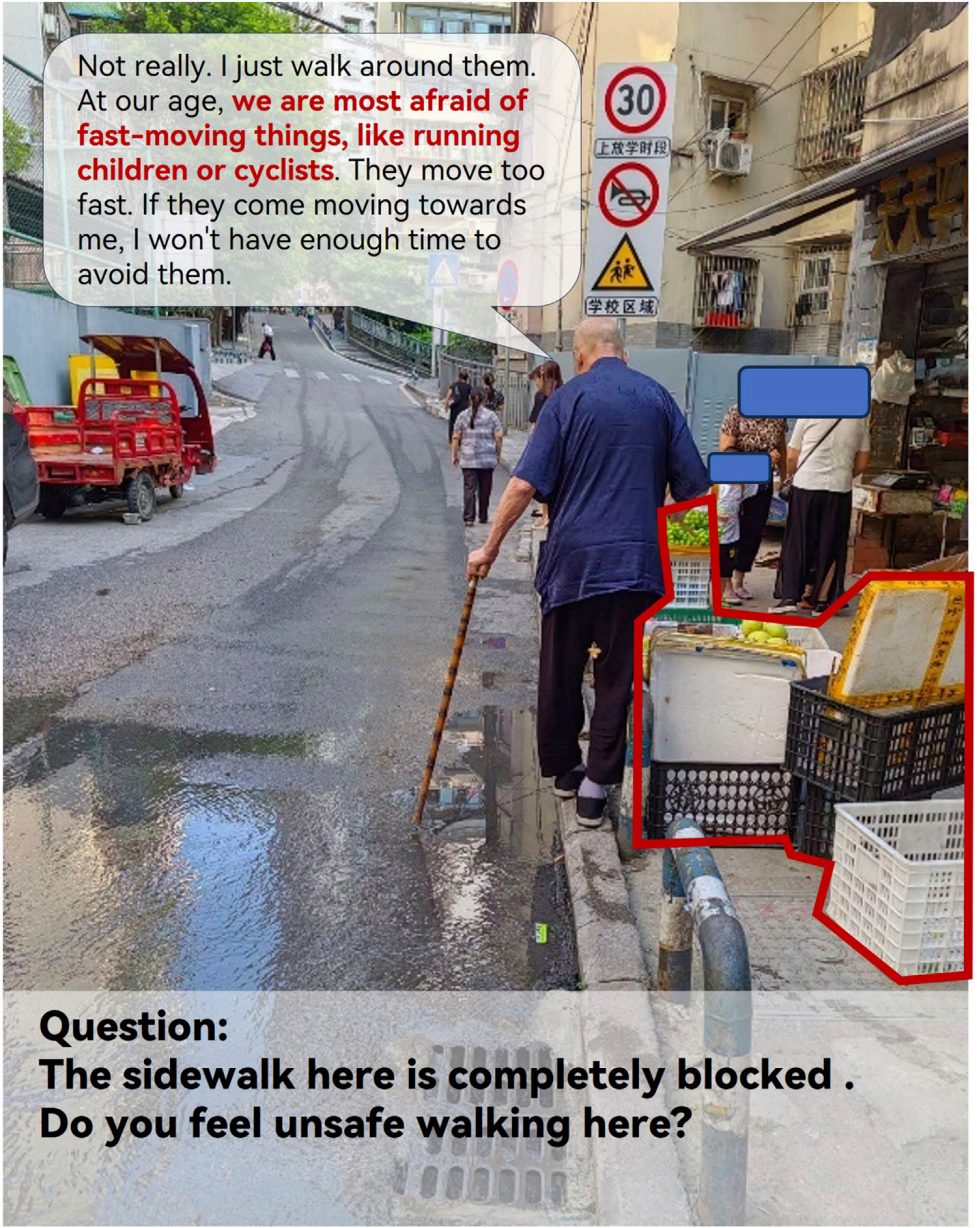
Participant avoiding obstacles (Chongqing, China. 2024).

Based on the interview text and accompanying photos, a noticeable disparity emerged between the participants’ verbal and nonverbal language when discussing their feelings about the sidewalk environment (Figure 4). To gain more insight, a conversation was initiated with the participant, who explained that they could slow down to avoid stationary obstacles. However, the participant expressed significant fear and concern regarding moving obstacles on the sidewalk. These findings contribute to a deeper understanding of the walking concerns of SSWS and can guide urban planners in prioritizing environmental improvements.

#### Sitting Facilities

Over a quarter of the participants expressed dissatisfaction with the lack of seating options in the neighborhood. Due to the scarcity of sitting facilities, individuals often have to continue walking until they reach their destination or find an alternative place to rest. Occasionally, they resort to utilizing other facilities for temporary seating, such as steps or guard post columns along the roadside. Notably, two participants mentioned that certain shops provide stools or chairs in front of their establishments, intended for public use. These informal sitting facilities support their mobility within the community.

#### Traffic Safety

A quarter of the participants expressed significant fear when it came to crossing the road. The challenges they encountered included a lack of crosswalks and traffic signals, short crossing signal durations, and heavy traffic. However, they perceived drivers in the neighborhood consistently maintained slow speeds, contributing to a sense of traffic safety. Two participants mentioned that when intending to cross a street, they often wave to drivers to ensure that approaching cars would stop for them.

Four participants expressed concerns about the use of personal mobility devices (e.g., bikes, electric bikes, and scooters) on sidewalks. They observed that these users often move quickly and sometimes go against traffic on sidewalks, giving rise to safety issues.

#### Weather

Weather conditions significantly impact the walking and mobility of SSWS. While 30.4% of the participants who discussed weather mentioned their inclination to stay indoors on rainy days due to slippery sidewalks caused by puddles, there are still few participants (*N* = 2) expressed a desire to go out and socialize even on rainy days. They expressed a wish for more sheltered spaces to facilitate outdoor activities during inclement weather.

The climate during the data collection period included warm weather as well as extremely hot weather. Participants indicated their willingness to adjust their travel behaviors to better accommodate extreme weather conditions, but they would not cancel their outings. They chose to go out during cooler times, reduce the frequency of outings, and shorten walking distances, demonstrating their strong ability to adapt to the environment. Additionally, participants emphasized the significance of environmental factors, such as the presence of trees and sheltered walkways, which offer shade and protection from rain, making walking more comfortable and feasible in different weather conditions.

Now I leave home at 8 o’clock in the morning, much earlier than before, to avoid the heat. When the weather is comfortable, I go out at least twice a day, but now I don't go out in the afternoon anymore because I’m worried about heatstroke. (Participant 01)

#### Trees and Greenery

Six participants expressed appreciation for the presence of trees and greenery in the neighborhood but also noted a shortage of them. They expected a more naturalistic environment, perceiving it as both relaxing and conducive to health.

#### Lighting

Few participants (11.1%) mentioned the impact of lighting on their mobility, as almost all SSWS (97.2%) do not participate in outdoor activities at night. One participant mentioned the broken solar-powered lights in the community, which made them feel that the environment was disorderly. Another participant complained about the dark stairwell of the residential building, which brings fear and uncertainty and affects their mobility, even during the day.

### Difference Between Demographic Groups

Overall, while the same environmental factors were discussed across various age and gender groups, significant differences emerged in the frequency with which certain environmental characteristics were discussed among demographic groups (see Supplementary Appendices B, C). Men were more inclined to express their opinions on environmental issues than women, with a heightened focus on the behavior of other sidewalk users, drivers’ behavior, and the impact of weather on outdoor activities. Younger seniors expressed more concern for daily facilities within walking distance, likely due to ongoing household responsibilities.

## Discussion

Despite an extensive body of research focusing on the relationship between slow walking and health outcomes in older adults [5, 7, 31, 32], this study stands out as the first to specifically examine the mobility and physical activity of SSWS within the built environment. All participants expressed their intention to engage in daily outdoor activities, even in extreme weather conditions, indicating a strong demand for outdoor activities within this group. Recognizing the mobility challenges encountered by SSWS is essential for designing inclusive built environments that cater to the needs of all individuals. This contributes to understanding the older population and complements the existing literature.

### Environmental Barriers and Facilitators for SSWS

The analysis revealed prominent themes related to the built environment, including topographical features, neighborhood services, sidewalk environment, sitting facilities, traffic safety, weather, trees and greenery, and lighting. While the findings align with prior research on perceptions of the built environment among the general older adult population [10, 33–37], participants explicitly mentioned additional subthemes related to the specific features of the built environment acting as barriers. Furthermore, some indicators have specific explanations for SSWS, which differ from those of the general older population and wheelchair users.

This study identified wet markets and supermarket as the most frequently visited destination for individuals with slow walking speeds, which aligns with Chinese consumers’ consumption habits and dietary culture [14, 39]. Therefore, providing facilities such as wet markets and vegetable markets and improving their environment is highly recommended as it is an essential aspect of creating an age-friendly environment in Chinese cities. Beyond the mere availability of daily destinations, participants emphasized the importance of specific features of these destinations. Four participants expressed concerns about the unsanitary conditions of the wet market, deeming it a potential safety hazard in their daily lives.

The findings of this study reveal that the majority of participants maintain a living radius of 400 m. Participants emphasized the importance of convenient access to daily facilities and resting areas in their immediate surroundings. The high frequency of older adults visiting daily facilities is driven not only by their daily needs but also by the opportunities they provide for social interactions [10, 16]. Easy access to destinations enhances the sense of security associated with the concept of “place” and contributes to a positive living experience [40]. A participant with limited mobility due to visual impairment stressed the importance of the building setback, emphasizing that it facilitates social interactions and street life observation. This aligns with previous studies [41, 42], which also highlight that the setback of buildings from the street positively impacts the wellbeing of older adults. We emphasize the significance of a nearby 400-meter home and building setback for SSWS, providing clear and actionable guidance for urban planners.

Stairs and ramps have consistently been identified as a major concern for older adults [10, 11, 15, 19, 33, 34]. For wheelchair users, stairs are considered significant obstacles and must be avoided due to the increased difficulty of walking [19]. The findings of this study indicate that slow walkers generally tolerate a few steps, and do not always opt for ramps over stairs, particularly when accessible routes involve much longer travel distances. However, the study reported that stairs with long flights and steep slopes should be avoided, as they significantly hinder walking, affecting not only mobility but also increasing the risk of falling.

Consistent with previous studies, participants valued the availability of handrails when navigating hills or stairs [15, 33, 44]. Nevertheless, handrails were often unavailable because they are absent, dirty, or incorrectly installed. These findings highlight the need to address numerous details in the practical implementation of universal design.

Another category of environmental factors that can be more easily modified is the sidewalk environment. Prior research has delved into the intricacies of sidewalk design to support outdoor activities for older adults. Additionally, consistent with the findings of Grant et al. [43] and Van Cauwenberg et al. [10], the results of this study revealed that a major concern for slow walkers regarding sidewalks was moving objects, such as careless cyclists. This highlights the importance of segregating sidewalks from cycling lanes.

Regarding traffic safety, street crossings posed significant challenges for participants, with issues including short crossing times, poorly marked crosswalks, obstructed views, fast-moving cars, and careless drivers being prominent discussion themes [33, 45]. An individual’s perceived sense of safety holds importance, with fast-moving car traffic as a major source of insecurity, similar to the fear experienced while walking on sidewalks. As a result, slow walkers generally avoid crossing arterial roads in their daily activities, as it is a significant barrier for them.

Aligned with previous studies [35, 46], this research highlights the significance of sitting facilities for SSWS. A seating area not only serves as a resting spot but also as a place for social gatherings [15]. Participants reported that the location of seats and benches was more important than their material or form. Therefore, it is important to provide a variety of seating options in urban areas. According to Jan’s research [47], well-situated primary seating should be complemented by numerous secondary seating options, allowing individuals to rest more informally and spontaneously, such as pedestals, steps, stones, bollards, fountains, or raised planters.

Urban greenery benefits social, ecological, physical, physiological health, and overall wellbeing, with focus on older adults’ positive impacts [48]. In urban communities, urban greenery plays an important role for individuals with slow walking speeds, supporting not only their physical activity but also providing a sense of relaxation. Additionally, hot days do not significantly impact outdoor physical activity, aligning with the findings of Lanza et al. [49]. The presence of trees and greenery becomes even more important as they can help improve the micro-climate of the neighborhood, providing shade and creating a more comfortable environment for older adults.

### Strengths and Limitations

This study focuses on the exploration of walking barriers and concerns within the demographic of slow walkers, shedding light on an often marginalized group with unaddressed health needs in age-friendly environment research. The utilization of go-along interviews allowed for the collection of rich, contextual data by integrating narrative accounts with geographical and visual elements, offering a micro-scale understanding of walking barriers and facilitators. Additionally, the study demonstrated the potential of go-along interviews in exploring the environmental perceptions of older adults, given their higher-level tolerance.

This study also has some limitations. Firstly, participant recruitment relied on intercept methods, potentially raising concerns about sample representativeness [17, 20, 23]. By exclusively focusing on SSWS who engage in outdoor activities, there is a potential oversight of those who may be unwilling or unable to go outdoors due to environmental constraints. Secondly, data collection was confined to a single city in China. Given that physical activity behaviors are affected by personal, social, and environmental factors [50], further research is needed to enhance the understanding of SSWS.

### Summary

The findings outlined the activity-travel patterns of SSWS, illustrating a concentration of their activities within a 400-meter radius of their homes. Eight categories of environmental themes (i.e., topography, neighborhood services, sidewalks, seating, traffic safety, weather, greenery, and lighting) that either supported or hindered the mobility of SSWS have been identified. Significant mobility barriers identified included long stairs, steep slopes, fast-moving objects on sidewalks, road crossings, and fast traffic. Supportive factors for their mobility included available handrails, nearby food-service places, ample seating, and greenery. The insights gained from this study hold significant implications for urban planners and policymakers. While striving for inclusivity is ideal, it is challenging to design for everyone, especially in developing countries [19, 51]. Therefore, it is recommended that general design frameworks and guidelines incorporate slow walkers rather than general adults as a benchmark. These improvements would not only benefit the general public but also enhance the mobility of slow walkers at a minimal cost. Ultimately, they foster increased physical activity for SSWS, promote their health, and potentially reduce healthcare costs.

## Data Availability

The interview data used during the study are available from the corresponding author by request.
