# Supplementary material for: “I Go Outdoors for Activities Every Day”: Go-Along With Seniors With Slow Walking Speeds to Explore Environmental Factors Influencing Mobility
Source: Int J Public Health. 2024 Jun 4;69:1607033. doi: 10.3389/ijph.2024.1607033 (PMC11182988; doi:10.3389/ijph.2024.1607033)
Supplement: Supplementary file 1 [file DataSheet1.docx]

**Appendix A: The walking distance and OD distance for the participants**

| **Number** | **Walking distance (m)** | **OD distance (m)** |
| --- | --- | --- |
| 1 | 847 | 300 |
| 2 | 231 | 113 |
| 3 | 267 | 214 |
| 4 | 436 | 139 |
| 5 | 1126 | 263 |
| 6 | 886 | 286 |
| 7 | 233 | 169 |
| 8 | 1082 | 182 |
| 9 | 124 | 106 |
| 10 | 395 | 118 |
| 11 | 1308 | 334 |
| 12 | 537 | 132 |
| 13 | 790 | 631 |
| 14 | 168 | 113 |
| 15 | 421 | 415 |
| 16 | 1444 | 399 |
| 17 | 380 | 261 |
| 18 | 170 | 105 |
| 19 | 175 | 170 |
| 20 | 157 | 123 |
| 21 | 167 | 44 |
| 22 | 413 | 409 |
| 23 | 271 | 74 |
| 24 | 57 | 36 |
| 25 | 376 | 265 |
| 26 | 987 | 369 |
| 27 | 283 | 98 |
| 28 | 427 | 343 |
| 29 | 266 | 190 |
| 30 | 181 | 167 |
| 31 | 748 | 218 |
| 32 | 894 | 500 |
| 33 | 666 | 310 |
| 34 | 504 | 283 |
| 35 | 631 | 70 |
| 36 | 377 | 150 |

**Appendix B: Percentages of gender groups that discussed an environmental category**

| **Environmental factors** | **Total** | **Male** | | **Female** | |
| --- | --- | --- | --- | --- | --- |
|  |  | **Count** | **%** | **Count** | **%** |
| Total | 36 | 13 | 100.0% | 23 | 100.0% |
| **Topographical features** | 32 | 11 | 84.6% | 21 | 91.3% |
| Stair/Step | 28 | 10 | 76.9% | 18 | 78.3% |
| Ramp | 14 | 6 | 46.2% | 8 | 34.8% |
| Handrail | 14 | 6 | 46.2% | 8 | 34.8% |
| **Neighborhood services** | 28 | 10 | 76.9% | 18 | 78.3% |
| Daily facilities | 18 | 7 | 53.8% | 11 | 47.8% |
| Rest areas | 16 | 4 | 30.8% | 12 | 52.2% |
| Public transport | 2 | 1 | 7.7% | 1 | 4.3% |
| Overall | 10 | 3 | 23.1% | 7 | 30.4% |
| **Sidewalk environment** | 18 | 7 | 53.8% | 11 | 47.8% |
| Sidewalk qualities | 17 | 6 | 46.2% | 11 | 47.8% |
| The behavior of other sidewalk users | 2 | 2 | 15.4% | 0 | 0.0% |
| **Traffic safety** | 19 | 9 | 69.2% | 10 | 43.5% |
| Crossings | 14 | 6 | 46.2% | 8 | 34.8% |
| Driver’s behavior | 5 | 4 | 30.8% | 1 | 4.3% |
| Others | 2 | 1 | 7.7% | 1 | 4.3% |
| **Weather** | 23 | 10 | 76.9% | 13 | 56.5% |
| Hot days | 16 | 6 | 46.2% | 10 | 43.5% |
| Rainy days | 9 | 5 | 38.5% | 4 | 17.4% |
| **Benches and sitting place** | 19 | 7 | 53.8% | 12 | 52.2% |
| The presence of a sitting place | 18 | 7 | 53.8% | 11 | 47.8% |
| Qualities | 3 | 1 | 7.7% | 2 | 8.7% |
| **Trees and greenery** | 6 | 3 | 23.1% | 3 | 13.0% |
| **Lighting** | 4 | 1 | 7.7% | 3 | 13.0% |
| Note: The percentage values are categorized into four groups to clearly illustrate the differences among subgroups: less than 25% (few), between 25% and 50% (some), between 50% and 75% (a lot), and more than 75% of the participants (almost all) (Van Cauwenberg et al., 2012). These categories are color-coded, with lighter shades representing lower percentages. | | | | | |

**Appendix C: Percentages of age groups that discussed an environmental category**

| **Environmental factors** | **Total** | **60~69** | | **70~79** | | **80~89** | | **＞90** | |
| --- | --- | --- | --- | --- | --- | --- | --- | --- | --- |
|  |  | **Count** | **%** | **Count** | **%** | **Count** | **%** | **Count** | **%** |
| Total | 36 | 8 | 100.0% | 13 | 100.0% | 12 | 100.0% | 3 | 100.0% |
| **Topographical features** | 33 | 7 | 87.5% | 12 | 92.3% | 11 | 91.7% | 3 | 100.0% |
| Stair/Step | 28 | 6 | 75.0% | 10 | 76.9% | 10 | 83.3% | 2 | 66.7% |
| Ramp | 14 | 3 | 37.5% | 6 | 46.2% | 3 | 25.0% | 2 | 66.7% |
| Handrail | 14 | 2 | 25.0% | 6 | 46.2% | 4 | 33.3% | 2 | 66.7% |
| **Neighborhood services** | 28 | 7 | 87.5% | 8 | 61.5% | 10 | 83.3% | 3 | 100.0% |
| Daily facilities | 18 | 4 | 50.0% | 7 | 53.8% | 7 | 58.3% | 0 | 0.0% |
| Rest areas | 16 | 3 | 37.5% | 4 | 30.8% | 6 | 50.0% | 3 | 100.0% |
| Public transport | 2 | 2 | 25.0% | 0 | 0.0% | 0 | 0.0% | 0 | 0.0% |
| Overall | 10 | 0 | 0.0% | 3 | 23.1% | 6 | 50.0% | 1 | 33.3% |
| **Sidewalk environment** | 18 | 6 | 75.0% | 5 | 38.5% | 7 | 58.3% | 0 | 0.0% |
| Sidewalk qualities | 17 | 5 | 62.5% | 5 | 38.5% | 7 | 58.3% | 0 | 0.0% |
| The behavior of other sidewalk users | 2 | 1 | 12.5% | 0 | 0.0% | 0 | 0.0% | 1 | 33.3% |
| **Traffic safety** | 19 | 4 | 50.0% | 7 | 53.8% | 7 | 58.3% | 1 | 33.3% |
| Crossings | 14 | 2 | 25.0% | 5 | 38.5% | 6 | 50.0% | 1 | 33.3% |
| Driver’s behavior | 5 | 1 | 12.5% | 1 | 7.7% | 3 | 25.0% | 0 | 0.0% |
| Others | 2 | 1 | 12.5% | 1 | 7.7% | 0 | 0.0% | 0 | 0.0% |
| **Weather** | 23 | 6 | 75.0% | 7 | 53.8% | 9 | 75.0% | 1 | 33.3% |
| Hot days | 16 | 4 | 50.0% | 5 | 38.5% | 7 | 58.3% | 0 | 0.0% |
| Rainy days | 9 | 3 | 37.5% | 2 | 15.4% | 3 | 25.0% | 1 | 33.3% |
| **Benches and sitting place** | 19 | 4 | 50.0% | 7 | 53.8% | 6 | 50.0% | 2 | 66.7% |
| The presence of a sitting place | 18 | 4 | 50.0% | 6 | 46.2% | 6 | 50.0% | 2 | 66.7% |
| Qualities | 3 | 0 | 0.0% | 1 | 7.7% | 1 | 8.3% | 1 | 33.3% |
| **Trees and greenery** | 6 | 2 | 25.0% | 2 | 15.4% | 2 | 16.7% | 0 | 0.0% |
| **Lighting** | 4 | 1 | 12.5% | 2 | 15.4% | 1 | 8.3% | 0 | 0.0% |
| Note: The percentage values are categorized into four groups to clearly illustrate the differences among subgroups: less than 25% (few), between 25% and 50% (some), between 50% and 75% (a lot), and more than 75% of the participants (almost all) (Van Cauwenberg et al., 2012). These categories are color-coded, with lighter shades representing lower percentages. | | | | | | | | | |
